# Effects of socioeconomic status on physical activity and cardiovascular diseases prior to and during the COVID-19 pandemic in the older adults

**DOI:** 10.3389/fpubh.2023.1241027

**Published:** 2023-09-11

**Authors:** Dongwoo Yang, Seo-Hyung Yang, Jae-Moo Lee, Jung-Min Lee, Jahyun Kim

**Affiliations:** ^1^Center for Regional Development, Chonnam National University, Gwangju, Republic of Korea; ^2^School of Global Sports Studies, Korea University, Sejong, Republic of Korea; ^3^College of Sport Science, Sungkyunkwan University, Suwon, Republic of Korea; ^4^Department of Physical Education, Kyung Hee University, Yongin, Republic of Korea; ^5^Department of Kinesiology, California State University Bakersfield, Bakersfield, CA, United States

**Keywords:** COVID-19, health inequalities, physical activity disparity, cardiovascular diseases, older population, Socioeconomic status, prescribed physical activity

## Abstract

**Purpose:**

This research seeks to evaluate the repercussions of socioeconomic status (SES) on physical activity (PA) among the older population, both pre and intra-COVID-19 pandemic. The study aims to scrutinize whether alteration in PA behaviors based on SES impacts cardiovascular diseases (CVDs). It is well established that PA has a significant association with CVDs and the pandemic has restricted PA in the older population. We endeavor to discern whether SES modulates PA levels and whether these levels of PA behavior subsequently influence the incidence of CVDs among older adults.

**Methods:**

The analytical framework of this study relies on the data procured from the Fact-Finding on the Status of Senior Citizens (FSSSC) survey conducted in 2017 and 2020, involving 10,299 (75 ± 6 years) and 10,097 (74 ± 6 years) participants, respectively. We employ Structural Equation Modeling (SEM) to elucidate the ramification of the COVID-19 pandemic on CVDs while accommodating potential mediating and confounding variables, including socioeconomic status, PA levels, body mass index (BMI), and gender, in the context of the pandemic and CVDs.

**Results:**

Our empirical models indicated a tendency for older adults of lower socioeconomic status (SES) to exhibit diminished levels of physical activity (PA) compared to their counterparts of higher SES, particularly considering the influence of the COVID-19 pandemic. Furthermore, prolonged engagement in PA is associated with a reduced risk of hypertension (*p* = 0.010), and congestive heart failure & arrhythmia (*p* < 0.001), when accounting for confounding factors.

**Conclusion:**

The COVID-19 pandemic has generated an SES-based disparity in PA among older adults, despite PA time being greater in older individuals with higher SES. Interestingly, this did not result in a reduction in CVDs. Therefore, the study emphasizes the need for targeted exercise programs may be necessary to mitigate health inequality among the older population.

## Introduction

The advent of the COVID-19 pandemic has necessitated the implementation of significant modifications in customary lifestyle routines and the configuration of the healthcare system, primarily due to the instigation of social distancing measures by authorities commencing in early 2020. Although these regulations have effectively decelerated the propagation of the virus, they have also been associated with potential adverse consequences on health, encompassing diminished accessibility to healthcare services (1). In particular, the older demographic manifests increased susceptibility to the virus and a higher propensity to develop exacerbated symptoms (2). Moreover, social distancing protocols have curtailed their social engagement and participation (3).

A majority of social engagements, particularly among older adults, is associated with physical activities or participation in sports (4). However, the Implementation of the lockdown policies during the COVID-19 pandemic has constrained such social and PA (5). Moreover, PA levels serve as a critical determinant of overall health in the older population, with social distancing measures potentially exerting the most significant influence on these levels (6). Previous studies substantiate a robust association between PA levels and cardiovascular diseases (CVDs) (7). CVDs account for one of the leading causes of mortality, with the annual expenditure related to these diseases surpassing $34 billion in the United States alone (8). Notably, individuals aged over 60 years exhibit inferior cardiovascular health relative to other population groups, and the mortality rate attributable to CVDs has escalated to 18.7% since 2010 (9). Consequently, the reduction in PA levels during the COVID-19 pandemic may adversely impact cardiovascular health in the older population, thereby engendering significant public health concerns.

Studies have demonstrated robust associations between socioeconomic status (SES) and both well-being and health (10). CVDs have been identified as the predominant cause of premature mortality in low- and moderate-income countries (11). Moreover, studies have elucidated that, compared to those with a lower SES, individuals of a higher SES manifest augmented levels of PA within the older population (12). Based on the previous literature, the higher CVD-induced mortality in low- and moderate-income countries may be influenced by PA level discrepancy. The social distancing regulations implemented amidst the COVID-19 pandemic have constrained access to PA facilities for the older population, and SES may facilitate disparate opportunities for accessing these facilities and participating in PA. As a result, the low SES group had less recreational PA during the COVID 19 pandemic (13). However, we do not know how much COVID-19 pandemic-induced PA discrepancy influenced on CVD confirmations in the older population during the COVID-19 pandemic.

Although the COVID-19 pandemic turned to endemic in 2023, the pandemic era provided a unique situation to investigate how much health discrepancy could be explained by SES-associated PA time. Investigating in how the COVID-19 pandemic impacted health discrepancies by SES-medicated PA time would provide opportunities to understand and develop public policies to reduce health discrepancies among different SES groups. Therefore, this study aims to examine the extent to which the COVID-19 pandemic has affected the disparity in PA levels as a function of SES within the older population and the subsequent contribution to the prevalence of CVDs. Our hypothesis postulates that the lockdown measures instituted as a response to the pandemic have engendered an SES-based disparity in PA levels based, subsequently amplifying the prevalence of CVD among the older population.

## Data and methods

### Sample data

Sample datasets were obtained from the Fact-finding Survey on the Status of Senior Citizens (FSSSC) conducted triennially by the Ministry of Health and Welfare (MoHW) of the Republic of Korea. The survey aims to collect various types of information from a randomized sample of senior citizens to present snapshots of the current status of senior citizens in South Korea, including general characteristics, the quality of relationships with people and communities, lifestyles, health status, and economic status. This study drew the FSSSC datasets from 2017 and 2020, comparing circumstances pre- and in-COVID-19. The FSSSC 2020 was conducted during the pandemic, from February 14 to November 20, 2020, involving 10,097 senior citizens aged 65 years or older. The FSSSC 2017 involved 10,299 seniors randomly selected and interviewed between June 8 and August 28, 2017 (14, 15).

### Variables used for SEM

#### Health risks and outcomes

Survey participants in the FSSSC for 2017 and 2020 were asked whether they had been diagnosed with four CVDs (hypertension, stroke, angina pectoris [AP], and myocardial infarction [MI]), and congestive heart failure (CHF) and arrhythmia for 12 months prior to the interviews. Responses were recorded dichotomously as 1 for “diagnosed” or 0 for “not diagnosed” for each type of disease. Only four CVD diagnostic variables related to cardiovascular health were used as dependent variables.

#### Participation in PA and BMI

Seniors reported the number of PA days per week and the average duration in minutes per PA as continuous independent variables. Multiplying the number of PA days per week and the PA minutes, we obtained a new variable, representing PA volume in minutes per week, which was used to evaluate the likelihood of being diagnosed with CVDs. In addition, Body Mass Index (BMI) was calculated for each survey participant using self-reported meters heights and body weights in kilograms.

#### Socioeconomic variable

We included an SES variable to examine its impact on PA volume and the likelihood of being diagnosed with CVDs. In the FSSSC dataset, we used the average annual household income data.

### Analytical model

We constructed structural equation models (SEM) to assess the indirect effects of SES on CVDs with SES – PA – CVD pathways and the direct effect, controlling BMI and gender. We constructed the SEMs for 2017 and 2020 and compared the magnitudes of effects of the SES variable on the CVDs between two periods of years.

**Figure 1:**
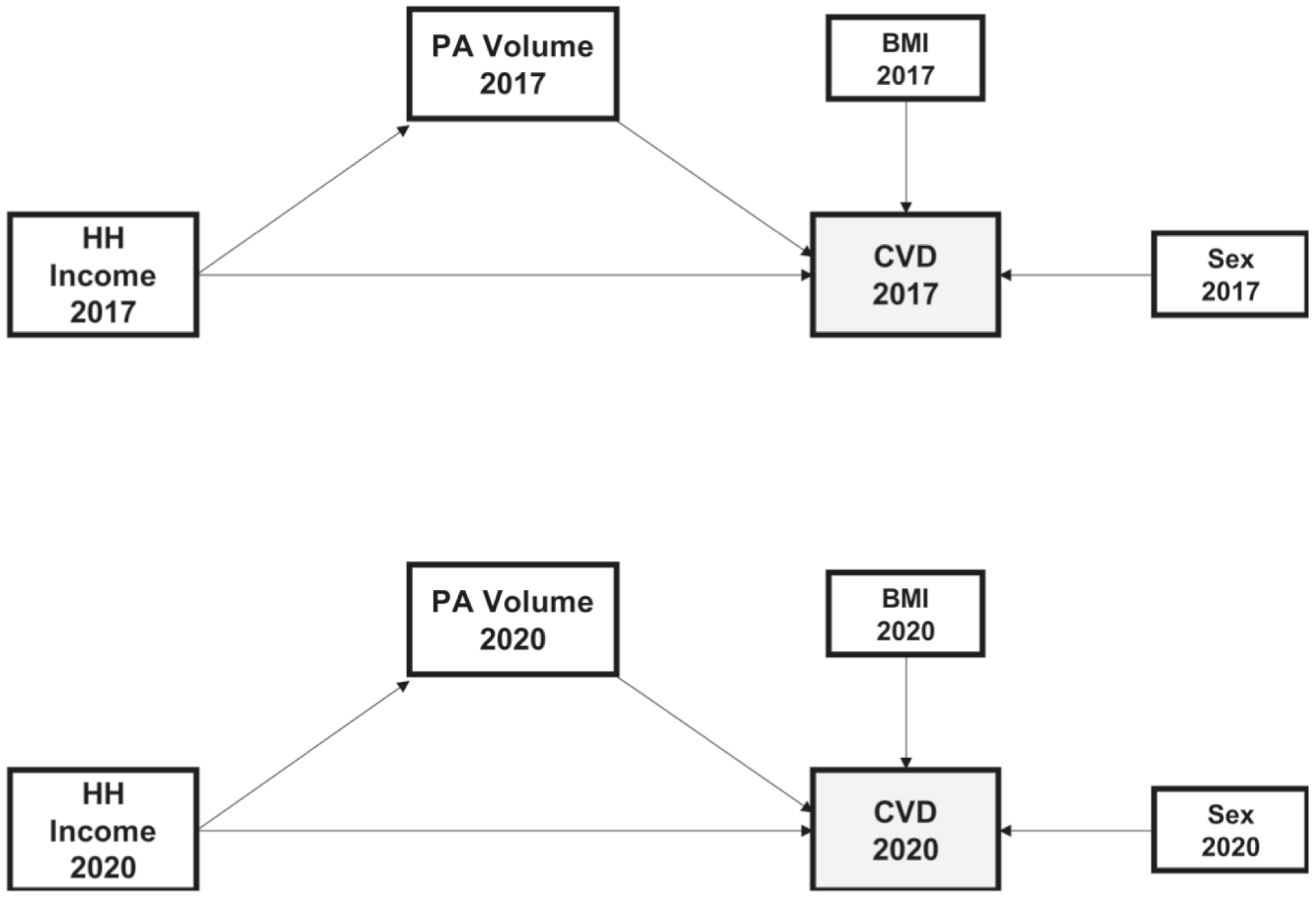
Model construction to predict the impacts of the PA Volume, BMI, Sex, and HH Income. The model is fitted from self-administrated data in the Fact-finding Survey on Status on Senior Citizens in 2017 and 2020. Single-headed arrows indicate the causal effect. HH Income, Household Income, logged; PA Volume, Physical Activity Volume (mins per week), logged; BMI, Body Mass Index (kg/m^2^); CVD, being diagnosed with CVD.

Figure 1 presents paths that support the hypothesis of this study. First, we investigated SES’s direct and indirect effects on CVDs for each survey period. We observed that PA volume had a direct influence on CVDs, and it acted as a mediator between SES and CVDs. Therefore, BMI was included as a confounding factor. Furthermore, we considered household income as an SES measure, both direct and indirect factors for PA and CVDs.

## Results

### Descriptive statistics

This study drew four CVD-related variables from the FSSSC 2017 and 2020 surveys, including diagnoses of hypertension, stroke, angina pectoris (AP) and myocardial infarction (MI), and congestive heart failure (CHF) and arrhythmia. The surveys found that in 2017, 59.5% of participants reported being diagnosed with hypertension; in 2020, that number was 57.6%. For stroke, the older adult group showed a lower risk rate during the pandemic (7.6% in 2017 compared to 4.1% in 2020). In addition, the diagnosis rates for AP & MI and CHF & arrhythmia were lower in 2020 than in 2017 (4.6% in 2020 versus 7% in 2017).

This study older population among the older population during the COVID-19 pandemic. The older population group averaged 173.9 mins of participating in PA per week in 2017, but in 2020, that number dropped to 121.5 mins per week.

In 2017, among the participants in the FSSSC, the percentage of males was 73.3%, while the percentage of females was 26.7%. In 2020, the ratio of males was 66.4%, and the ratio of females was 33.6%. In the 2017 survey, the average BMI of the participants was 23.5, while in 2020, it was 23.6. It was observed that there was no significant difference in BMI between the two periods (Table 1).

**Table 1:** Descriptive statistics of research variables in the years 2017 and 2020.

Characteristic	2017		2020	
	Freq. (%)	Mean ± S.D.	Freq. (%)	Mean ± S.D.
**Risk**				
Diagnosed – Hypertension	6,126 (59.5)		5,818 (57.6)	
Diagnosed – Stroke	787 (7.6)		417 (4.1)	
Diagnosed – AP & MI	748 (7.3)		460 (4.6)	
Diagnosed – CHF & Arrhythmia	732 (7.1)		463 (4.6)	
**Physical activity**				
Workout Minutes per Week		173.9 ± 209.4		121.5 ± 170.5
**Anthropometric**				
Male	7,547 (73.3)		6,707 (66.4)	
BMI (kg/m^2^)		23.5 ± 3.1		23.6 ± 2.6
**Socioeconomic status**				
HH Income (10 K KRW)		2,419.4 ± 2,093.2		2,700.9 ± 3,954.1

10 K KRW, Equivalent to 8 USD; AP, Angina Pectoris; MI, Myocardial Infraction; CHF, Congestive Heart Failure.

The economic measure associated with household economic status were also considered in this study. The FSSSC found that the average older population experienced better economic situations during the pandemic. Senior households’ economic situation was slightly better off in 2020 than in 2017. The average annual household income of the participants in 2020 was 27 million Korean Won, higher than in 2017 (24 million KRW). Since the COVID-19 emergency relief funds were distributed to households, economic situations during COVID-19 have temporarily improved (16).

### Results from the four CVD-models

Table 2 presents the empirical predictions of SEM models for four CVDs, separated by 2017 and 2020. A total of 10 SEMs were estimated by running four SEMs for each of the two years. Additionally, Table 2 demonstrates the direct effects among the variables. Due to the moderating effect of PA between SES and CVD, SES provides both direct and indirect effects on CVD. The direct and indirect effects of SES on CVD are summarized separately in Table 3.

**Table 2:** SEM estimations by CVDs for the older population in 2017 and 2020.

Risk	Pathways between variables (direct effects)			2017 Model		2020 Model	
				Coeff.	Z-value	Coeff.	Z-value
Hypertension	HH Income	→	PA Volume	0.224	6.404 ***	0.420	13.912 ***
	PA Volume	→	Hypertension	−0.008	−4.290 ***	−0.004	−2.277 *
	HH Income	→	Hypertension	−0.040	−5.508 ***	−0.055	−9.233 ***
	BMI	→	Hypertension	0.028	18.094 ***	0.023	12.107 ***
	Sex	→	Hypertension	−0.047	−4.036 ***	−0.043	−4.004 ***
Stroke	HH Income	→	PA Volume	0.224	6.404 ***	0.420	13.912 ***
	PA Volume	→	Stroke	−0.005	−4.765 ***	−0.001	−1.068
	HH Income	→	Stroke	−0.012	−3.094 **	−0.012	−4.894 ***
	BMI	→	Stroke	0.001	1.543	0.000	−0.081
	Sex	→	Stroke	0.014	2.137 *	0.002	0.461
AP & MI	HH Income	→	PA Volume	0.224	6.404 ***	0.420	13.912 ***
	PA Volume	→	AP & MI	−0.001	−1.023	0.000	0.017
	HH Income	→	AP & MI	−0.007	−1.729	−0.009	−3.611 ***
	BMI	→	AP & MI	0.004	4.242 ***	0.003	3.218 **
	Sex	→	AP & MI	0.007	1.051	0.002	0.503
CHF & Arrhythmia	HH Income	→	PA Volume	0.224	6.404 ***	0.420	13.912 ***
	PA Volume	→	CHF & Arrhythmia	−0.004	−3.903 ***	−0.001	−1.700
	HH Income	→	CHF & Arrhythmia	−0.005	−1.395	−0.007	−2.583 **
	BMI	→	CHF & Arrhythmia	0.000	0.334	0.001	1.454
	Sex	→	CHF & Arrhythmia	−0.025	−4.026 ***	−0.015	−3.248 **

Single-headed arrows indicate a direct effect from the variables on the left-hand side to those on the right-hand side.

Coeff., Coefficients; AP, Angina Pectoris; MI, Myocardial Infraction; CHF, Congestive Heart Failure; HH Income, Household Income; PA Volume, Physical Activity Volume (mins per week); CVD, Cardiovascular Disease; BMI, Body Mass Index (kg/m^2^); *** *p* < 0.001, ** *p* < 0.025, * *p* < 0.050.

**Table 3:** SEM estimations by CVDs for the older population in 2017 and 2020.

	Pathways between factors					Coefficients	
Effect type	SES		PA		Risk	2017 Model	2020 Model
Direct	HH Income		→		Hypertension	−0.040	−0.055
Indirect	HH Income	→	PA Volume	→	Hypertension	−0.002	−0.002
Direct	HH Income		→		Stroke	−0.012	−0.012
Indirect	HH Income	→	PA Volume	→	Stroke	−0.001	–
Direct	HH Income		→		AP & MI	–	−0.009
Indirect	HH Income	→	PA Volume	→	AP & MI	–	–
Direct	HH Income		→		CHF & Arrhythmia	–	−0.007
Indirect	HH Income	→	PA Volume	→	CHF & Arrhythmia	−0.001	–

Only statistically significant coefficients are included in the table; The sign ‘–‘represent no statistical significance. SES, Socioeconomic Status; PA, Physical Activity; AP, Angina Pectoris; MI, Myocardial Infraction; CHF, Congestive Heart Failure; HH Income, Household Income; PA Volume, Physical Activity Volume (mins per week).

#### Direct effect of SES on PA volume

HH income positively affected PA volume for 2017 and 2020 (Table 2). The effect of HH income on PA Volume during COVID-19 was about two times greater than in 2017. During COVID-19, seniors with higher SES tended to participate in PA more than in 2017.

#### Direct effect of PA volume on CVDs

It has been confirmed that PA Volume has a negative direct effect on hypertension. However, during the COVID-19 period, this effect’s strength was lower than before COVID-19 (Table 2). As for the relationship between PA Volume and stroke, higher PA Volume was found to lead to a lower risk of stroke, but this association was significant only in the 2017 model (Table 2).

#### Direct and indirect effects of SES on hypertension

After controlling for other variables, we discovered that higher HH income directly affects the lower risk of a hypertension diagnosis in 2017 and 2020. Comparing the magnitudes of the coefficients between the 2017 and 2020 models, the direct effect of HH income on hypertension during COVID-19 was more substantial than in 2017.

Considering the mediating effects of PA volume between SES and Risk, we found that higher HH income also indirectly affected a lower risk of hypertension. In contrast, there is no difference in the magnitude of the indirect effects between 2017 and 2020. The finding that there is no difference in the indirect effect of HH income between 2017 and 2020 contradicts the hypothesis of this study, which proposes that during the COVID-19 period, a higher SES would induce an increase in physical activity (PA) to reduce risk further (Table 3).

#### Direct and indirect effects of SES on stroke

Higher HH income influences lower stroke diagnoses. Negative causality was observed in both 2017 and 2020. However, the magnitude of the negative impact of HH income on stroke was consistent between 2017 and 2020.

When considering the mediating effect of PA volume between HH income and stroke, the indirect effect of HH income on stroke was found to be negative in 2017. However, during the COVID-19 period, we observed no significant indirect effect. Therefore, we fail to support the hypothesis that older adults belonging to higher SES during COVID-19 would engage in more PA and consequently reduce their risk (Table 3).

#### Direct and indirect effects of SES on AP and MI

Overall, it was found that SES has little influence on AP (angina pectoris) and MI (myocardial infarction). In the 2020 model, higher HH income directly reduced the risk of AP and MI. However, no significant indirect effect of SES on AP and MI was observed (Table 3).

#### Direct and indirect effects of SES on CHF and Arrhythmia

In the case of CHF (congestive heart failure) and arrhythmia, the direct impact of SES was not found in 2017, but in 2020. In 2020, it was observed that higher HH income induces a lower risk. Regarding the indirect effect mediated by PA volume, in 2017, a negative causality of SES on CHF and arrhythmia was found, but no significant indirect effect was found in 2020 (Table 3).

## Discussion

This study was designed to examine the effect of the COVID-19 pandemic on CVDs within the older population of different SES, attributing to a discrepancy in PA. The hypothesis was that SES impacts PA levels and this influence would be amplified during the COVID-19 pandemic period, with such PA level discrepancy contributing to the prevalence of CVDs in older adults. The results revealed a significant inverse relationship between higher household income and the incidence of hypertension, stroke, AP, MI, CHF, and arrhythmia during the pandemic period. Moreover, the effect magnitude of household income on CVDs is proved to be stronger than that observed pre-pandemic. The pandemic additionally elicited a decline in PA levels within the older population of lower SES compared to the pre-pandemic period (2017), although this discrepancy in PA by SES was not concomitant with an increased diagnosis of hypertension, stroke, AP, MI, CHF & arrhythmia.

The older population is subject to an elevated risk of cardiovascular diseases (CVDs) including MI, stroke, and coronary artery disease, precipitated by the incidence of hypertension, CHF & arrhythmia (8, 17–21). Hypertension is affiliated with arteriosclerosis, a pathological condition that can culminate in MI and CHF (17, 21). CHF typically ensues from either arteriosclerosis induced by hypertension or left ventricle hypertrophy, resulting in inadequate blood circulation throughout the organism (19, 22, 23). Additionally, arrhythmia, characterized by an aberration in the normal cardiac rhythm, can potentially engender conditions such as fatigue, dizziness, chest pain, cardiomyopathy, dementia, stroke, and cardiac arrest (20, 24, 25). Although those conditions may not instantly precipitate life-threatening CVD events and other complications at a later stage, besides inducing further complications in the older population (8, 18, 20). Moreover, this aggregation of biological, behavioral, and psychosocial risk factors, exceptionally heightened in those of lower SES, could potentially result in the onset of future CVD in the future (26).

SES demonstrates a direct association with the diagnoses of CVDs, attributable to heightened risk factors such as hypertension, obesity, diabetes, and physical inactivity inherent in this population (27, 28). These amplified risk factors contribute to an increased incidence of MI, all caused mortality, and cardiovascular-cause mortality in individuals of lower SES (29). Moreover, SES represents an independent predictor of CVD risk, distinct from traditional risk factors encompassed within the Framingham risk score for coronary heart disease (30). The results obtained from our current study unveil that the COVID-19 pandemic increased CVD diagnosis within the lower SES group. During the pandemic period, lower SES group has encountered constraints in accessing healthcare providers (1), potentially exacerbating the adverse consequences of CVDs within this population.

Physical inactivity, one of the risk factors contributing to the development of CVDs, exhibits a higher prevalence among lower SES group (27, 28). Furthermore, SES serves as a factor in accounting for this discrepancy in PA within the older population (31). We observed SES-based discrepancy in PA duration among the older population both pre-pandemic and amid the COVID-19 pandemic. Higher SES was correlated with increased PA levels in the old population both before and during the COVID-19 pandemic (Table 2). Moreover, the discrepancy in PA duration between high and lower SES groups widened during the pandemic (Table 2). This observation can be rationalized by varying accessibility to PA facilities dependent on SES. During the pandemic, indoor facilities like gyms and community centers were closed, limiting individuals to open spaces such as recreational parks. Areas with higher household income often exhibit fewer physical impediments to walking and greater access to public open spaces, such as walking and cycling trails (32). Moreover, the decrease in PA levels in the older population during the COVID-19 pandemic might be attributable to restricted access to PA facilities (5, 33). The lack of community open spaces in lower socioeconomic areas could elucidate the reduced PA duration among the economically disadvantaged elderly population in those areas during the pandemic.

Regular participation in PA has been found to considerably influence CVDs in the older population (34). Previous research also indicates a correlation between PA and hypertension, alongside CHF & arrhythmia (35–37). In 2017, our findings demonstrated that a discrepancy in PA duration as a result of SES elevates the incidence of hypertension, stroke, and CHF and arrhythmia (as seen in indirect effects in Table 3). Intriguingly, these CVD diagnoses did not show an escalation during the pandemic period despite the strong influence of SES on PA duration discrepancy (refer to indirect effects in Table 3). This unexpected outcome could potentially be explained by the intensity of exercise. The intensity of Exercise has been identified as a contributing factor to the incidence of CVD (38–40). Yu and colleagues showed that lower intensity leisure time physical activity (LTPA) such as walking or golfing does not demonstrate a significant association with mortality due to CVD. In contrast, high-intensity LTPA such as jogging and stair climbing effectively reduced the mortality linked to CVDs. Furthermore, this study also revealed that job-related physical activity does not attenuate mortality induced by CVDs (40), and comparable results were observed in the older population (39). Consequently, the intensity of exercise becomes a critical factor in evaluating the effects of PA duration discrepancy on the prevalence of CVDs in the older population within the higher SES. Despite the higher SES group engaging in a longer duration of PA during the pandemic, such high PA durations do not guarantee a significant attenuation in CVD diagnoses without the appropriate intensity of PA in the older population.

The physiological effects on the cardiovascular system vary with different intensities of PA. A plausible explanation for this phenomenon may be associated with the balance between reactive oxygen species (ROS) and nitric oxide (NO) (41). An upsurge in ROS within the vascular endothelial and smooth muscle cells leads to vascular endothelial dysfunction and initiates an inflammatory process. When this imbalance manifests, the ROS level escalates, resulting in cellular and tissue damage, alongside causing cardiovascular dysfunction and inflammation (42). On the other hand, an increment in NO levels results in a reduction in ROS levels, thereby ameliorating cardiovascular functions (43). Furthermore, one previous study has illustrated that mild intensity exercise (25% of VO_2_ max) does not confer beneficial impacts on oxidative stress markers and vascular function. However, moderate-intensity exercise (50% of VO_2_ max) enhances vascular function *via* nitric oxide synthase (NOS) activation and concurrently diminishes oxidative stress markers (44). The shear stress serves as a pivotal factor in elucidating this outcome. A study by Thijssen et al. (45) showed that shear stress intensifies with increasing intensity of exercise. Given that shear stress on the endothelial cells primarily stimulates the production of NO and NOS (46), lower levels of shear stress concomitant with less intensity physical activity may not yield beneficial effects, less NO production, on the cardiovascular system.

The lockdown period during the COVID-19 pandemic has been associated with a decline in PA levels among the general older population (5), a finding further confirmed by our current study. Moreover, our study has identified an SES-dependent variation in a decrease of PA among the older population. This reduction in PA duration was linked to a rise in diagnoses of hypertension, stroke, MI, CHF, and arrhythmia among older adults with lower SES prior to the pandemic (2017). However, an increased duration of PA among higher SES groups during the COVID-19 lockdown period did not result in a decrease in these conditions nor the prevalence of CVDs, an outcome that contradicts our initial expectations. This unanticipated result may be attributable to the intensity of the PA. Despite the higher levels of engagement in PA observed among the higher SES group, the intensity of these activities may have been insufficient to elicit favorable effects on the cardiovascular system. Before the COVID-19 lockdown period, the higher SES group not only had greater access to the PA facility but also had more opportunities to partake in designed or prescribed physical activity designed to stimulate the cardiovascular system. Several interventions have attempted to devise exercise programs suitable for the lower SES population to alleviate health inequality, However, the benefits of physical activity have proven inconsistent (47). Future studies may necessitate the development of structured PA programs designed specifically for the lower SES population, to reduce this health inequality across SES levels among the older population.

## Conclusion

Three years into the COVID-19 pandemic, the disease appears to be transitioning into an endemic status. Due to increased susceptibility, older population remain at risk and should exercise caution in crowded environments such as public gyms or community centers. According to our data, older adults of higher income have not exhibited PA duration changes compared to pre-pandemic levels; this observation could be attributed to the increased availability of open spaces for activities with wealthier communities. The accessibility to these public spaces may contribute to the maintenance of regular PA duration among older adults of higher SES during the COVID-19 pandemic. However, a longer duration of PA does not necessarily associate with improved health outcomes among higher socioeconomic older adults. This outcome can be potentially explained by the intensity of PA. With the gym and other physical activity facilities shuttered down during the pandemic, the higher SES group may face limitations in accessing their prescribed moderate-intensity exercise programs. This restriction could influence the prevalence of CVD prevalence during the pandemic period, as observed in our current study.

Our findings indicate a discrepancy in PA duration across SES levels. While the pandemic has widened this discrepancy, the intensity of PA might be a critical factor in reducing CVD prevalence in older adults. Consequently, it is crucial to take into account the intensity of physical activity to address health inequality among the older population, emphasizing the promotion of prescribed moderate-intensity PA. Further investigations are required to devise physical activity programs of appropriate intensity to diminish the prevalence of CVDs among the older populations with lower SES brackets.

## Strength and weakness

The strength of this study is that we used an SEM model to explain how PA duration discrepancy across SES levels on CVD prevalence among the older population. With this method, we were able to distinguish the direct and indirect effects of SES on CVD prevalence. Since the literature shows the association of SES with PA and the direct effect of PA on CVD, we focused on the PA as a mediator between SES and CVD. This enables model specification to be less biased than regression-related empirical models. The limitations of this study were that we conducted using pre-existed data. In this data, we had limited control over the survey questions. Furthermore, the pre-existed data did not encompass questions regarding the intensity of PA within this population. Therefore, the current evidence might be inadequate to corroborate our conclusion that the intensity of physical activity is a significant factor to explain this unexpected finding. These limitations may curtail the conclusiveness of our study. However, they also provide direction for future research endeavors aimed at reducing health inequality in the older population. This study presents another limitation to being cross-sectional research since the empirical analysis was conducted based on datasets at single points in time, such as the years of 2017 and 2020. Although the cross-sectional SEM provides potential causal relationships between variables, future research needs to combine cross-sectional SEM with longitudinal analyzes, to strengthen the evidence for causality.

## Data availability statement

The original contributions presented in the study are included in the article/supplementary material, further inquiries can be directed to the corresponding author.

## Ethics statement

The studies involving human participants were reviewed and approved by the study used anonymized and deidentified data on 2017 and 2020 FSSSCs as government-approved statistical surveys (Approval number: 117071). The participants provided their written informed consent to participate in this study. The studies were conducted in accordance with the local legislation and institutional requirements. Written informed consent for participation was not required from the participants or the participants' legal guardians/next of kin in accordance with the national legislation and institutional requirements.

## Author contributions

DY, J-MiL, and JK contributed to the conception and design of the research and interpreted the data. DY collected and analyzed data. DY and JK drafted the manuscript. DY, SY, J-MoL, J-MiL, and JK edited and revised the manuscript. All authors contributed to the article and approved the submitted version.

## Funding

This research was funded by California State University, Bakersfield (Grant, Research, and Sponsored Program and Teaching and Learning Center).

## Conflict of Interest

The authors declare that the research was conducted in the absence of any commercial or financial relationships that could be construed as a potential conflict of interest.

## Publisher’s note

All claims expressed in this article are solely those of the authors and do not necessarily represent those of their affiliated organizations, or those of the publisher, the editors and the reviewers. Any product that may be evaluated in this article, or claim that may be made by its manufacturer, is not guaranteed or endorsed by the publisher.

## Abbreviations

CVDs: cardiovascular diseases
SES: socioeconomic status
PA: physical activity
AP: angina pectoris
MI: myocardial infraction
CHF: congestive heart failure
FSSSC: Fact-Finding Survey on the Status of Senior Citizens
SEM: structural equation modeling
